# How shared is decision‐making in multidisciplinary tumour conferences with patient participation? An observational study

**DOI:** 10.1111/hex.13638

**Published:** 2022-10-31

**Authors:** Barbara Schellenberger, Christian Heuser, Annika Diekmann, Nicole Ernstmann, Anna Schippers, Franziska Geiser, Ingo G. H. Schmidt‐Wolf, Isabelle Scholl, Lena Ansmann

**Affiliations:** ^1^ Department for Psychosomatic Medicine and Psychotherapy, Center for Health Communication and Health Services Research (CHSR) University Hospital Bonn Bonn Germany; ^2^ Center for Integrated Oncology (CIO) University Hospital Bonn Bonn Germany; ^3^ Department for Psychosomatic Medicine and Psychotherapy, Faculty of Medicine University Hospital Bonn Bonn Germany; ^4^ Department of Integrated Oncology University Hospital Bonn Bonn Germany; ^5^ Department of Medical Psychology University Medical Center Hamburg‐Eppendorf Hamburg Germany; ^6^ Department of Health Services Research, School of Medicine and Health Sciences, Division for Organizational Health Services Research Carl von Ossietzky University Oldenburg Oldenburg Germany

**Keywords:** breast cancer, gynaecologic cancer, multidisciplinary tumour board, multidisciplinary tumour conference, oncology, patient‐centred communication, shared decision‐making

## Abstract

**Background:**

In some breast and gynaecologic cancer centres in Germany, patients participate in their own case discussion in multidisciplinary tumour conferences (MTCs), where treatment recommendations are discussed and finalized. However, the extent to which patients in MTCs are involved in decision‐making on treatment recommendations remains largely unexplored. Hence, this study investigates how recommendations are communicated to patients and the extent to which the interactions with patients in MTCs are in line with shared decision‐making (SDM).

**Methods:**

In this observational study, we audio‐recorded MTCs with patient participation in three breast and gynaecologic cancer centres in Germany. We qualitatively analysed the data with regard to content and linguistic aspects.

**Results:**

We analysed 82 case discussions. Recommendations made during MTCs were regarding (i) treatment options, (ii) treatment initiation, (iii) next (treatment) steps and (iv) whether a treatment method should be initiated at all. The decision about recommendations depended in part on patients' preferences or further course/further outcomes. Although the purpose of MTCs is to provide recommendations, some recommendations were framed as the final decision. The majority of the decision‐making conversation could be characterized as option talk (78%), during which patients were mostly proposed only one (treatment) option.

**Conclusions:**

This study establishes limited SDM in MTCs with patient participation. By indicating choices and thereby creating awareness of choices among patients, MTCs with patient participation could be used to foster SDM implementation.

**Patient or Public Contribution:**

Two representatives of a large self‐help organization for patients with breast cancer assisted the research project, particularly, in discussing the results.

## 1 BACKGROUND

Patients diagnosed with cancer must make complex and far‐reaching decisions regarding the treatment course together with multiple healthcare professionals.^1^ Shared decision‐making (SDM), in which there is a two‐way exchange of information, both parties reveal their treatment preferences, and then make a joint decision based on the information,^2^ is advocated primarily for preference‐sensitive decisions.^3^ Patients who are involved in decision‐making are more satisfied and have fewer decision conflicts.^4^ Besides, SDM is mostly preferred by patients and healthcare professionals.^5^, ^6^, ^7^, ^8^, ^9^, ^10^ For example, most patients with ovarian cancer (about 60%) select SDM with the healthcare professional, whereas approximately 15% want to make decisions autonomously, and about 20% want the healthcare professional to make the decision.^11^ Similarly, most patients with breast cancer prefer SDM (75%) for treatment decision‐making in general.^10^ However, SDM is often not implemented in routine care in oncology.^12^, ^13^, ^14^ Studies on outpatient oncology reported that only half of the patients feel that they have been offered treatment options.^15^ Other study results show that a large proportion of patients with breast cancer who preferred SDM (95.3%), but also a large proportion of patients who preferred physician‐led decision (93.7%), reported an intermediate or high degree of SDM.^10^

Organizational‐ and system‐level characteristics like teamwork and culture can influence SDM implementation.^16^, ^17^ Problems include the identification of preferences and their documentation, but also a noisy environment and lack of private space to talk with patients.^17^, ^18^ From the patients' standpoint, barriers to SDM include poor healthcare professional communication. SDM is facilitated when healthcare professionals consider patients' preferences, and patients experience healthcare professional behaviours that build rapport and trust.^19^ From healthcare professionals' standpoint, there are other barriers—healthcare professionals follow different definitions of SDM and hold different views on the extent of patient involvement and when SDM should be used. In the case of SDM, family involvement, good healthcare professional–patient relationships and informed patients are viewed as facilitators. However, a lack of (multidisciplinary) communication reportedly correlates with negative SDM experiences.^13^

Multidisciplinary care is a crucial principle of cancer care.^20^ In a multidisciplinary tumour conference (MTC), participating healthcare professionals meet regularly to discuss diagnosis and treatment options and to decide on recommendations based on the currently available evidence.^21^, ^22^ A multidisciplinary approach to treatment benefits both patients and healthcare professionals.^20^, ^23^, ^24^, ^25^ From the healthcare professionals' viewpoint, about three‐quarters of the MTC's opinions are perceived as recommendations and one‐quarter as decisions.^23^ A recommendation might denote a treatment method, the start of therapy, the next steps in treatment or whether therapy should be initiated at all.^26^

In MTCs, recommendations are largely based on biomedical information, building less on psychosocial information, comorbidities and preferences.^14^, ^27^, ^28^, ^29^, ^30^ Perhaps, the absence of information on psychosocial factors could be accountable for the lack of implementation of MTC recommendations.^14^, ^31^, ^32^, ^33^ Among other benefits, patient participation in their own case discussion might prevent this delay. In different oncology settings—MTCs, ward rounds and outpatient clinics—differences in decision‐making processes reportedly correlate with the presence or absence of patients.^27^ Despite studies available on the participation of patients in MTCs in Germany and Australia,^8^, ^34^, ^35^, ^36^ 5%–7% of patients in breast cancer care are known to participate in Germany.^21^, ^35^, ^37^

Participation itself is implemented in different ways in German cancer centres so that in some centres patients are present during the entire case discussion, and in other centres, patients are only allowed to participate in MTCs after treatment recommendations have been finalized.^34^ Previous research revealed both advantages and disadvantages of participation for healthcare professionals and patients.^8^, ^35^, ^38^

Previously, we explored how healthcare professionals weigh the opportunities and limitations of SDM in MTCs. We interviewed healthcare professionals with and without experience with patient participation in MTCs. According to healthcare professionals, some steps of SDM can be implemented in MTCs, while others can only be implemented partially or not at all, such as ‘disclosure that a decision needs to be made’.^39^ Even if guidelines indicate a preferable treatment option, how this option is discussed with patients could be more or less consistent with the principles of SDM. However, it remains unclear how recommendations are communicated to patients and whether SDM is practised in MTCs with patient participation. Hence, this study aims to fill this research gap and generate findings regarding recommendations and SDM in MTCs with patient participation. The research questions addressed in this study are as follows: (a) How are recommendations communicated to patients in MTCs? (b) To what extent are the interactions with patients in MTCs in line with SDM?

## 2 MATERIALS AND METHODS

### 2.1 Study design

The study presents results from a substudy of the multicenter, observational study ‘Patient involvement in multidisciplinary tumour conferences in breast cancer care—an exploratory study’ (PINTU^40^), in Germany's most populated state, North Rhine‐Westphalia. It was approved by the Ethics Committee of the Medical Faculty of the University of Cologne, Germany (Reference number for approval: 17‐405). This study was conducted at six breast and gynaecologic cancer centres, one of which included two hospitals.

### 2.2 Sample

We obtained data for the present substudy from three breast and gynaecologic cancer centres (*n* = 4 hospitals), in which patients were regularly invited to participate in MTCs. Before data collection, the research team visited hospitals and MTCs to explain the observation method. Medical staff on the wards recruited patients whose cases were to be discussed at an MTC. All eligible patients with breast and/or gynaecologic cancer were invited to the MTC at the hospitals where patients regularly attended MTCs. If they decided to participate in an MTC, they were invited to participate in our study. The inclusion criteria included those aged 18 years and above and diagnosed with breast and/or gynaecologic cancer (ICD‐10 codes C50.xx‐C58.xx, D05.xx‐D07.xx). We obtained written informed consent from all MTC participants and recruited patients.

### 2.3 Data collection

Data were collected from November 2018 to February 2020. A group of researchers (sociologist, psychologist, medical student, health services researcher, speech therapist) conducted passive participant observations, two of them during each MTC. Field notes included information on the number of healthcare professionals present, seating arrangement and length of each case discussion. Audio data from the case discussions were transcribed verbatim and anonymized.

For the PINTU study patients were asked to complete questionnaires: before the MTC (T0), immediately after the MTC (T1) and four weeks after the MTC (T2).^40^ To explain the sample, we used the T0 questionnaire data, combined with information from the case discussion and/or tumour documentation (cancer entity—breast and/or gynaecology, UICC staging). The sociodemographic data included age, marital status, having children, living in a partnership and level of education.

### 2.4 Analysis of communication

To investigate SDM in case discussions in MTCs with patient participation, we performed a qualitative content and linguistic analysis of the transcribed audio recordings of the case discussions. The software MAXQDA supported data analysis. Two researchers coded the expressions independently. For validation, identified codes were discussed and agreed upon in regular meetings within the research team. The first step was to identify whether a recommendation was made and which of the four types (treatment method, start of therapy, next steps in treatment or whether a treatment should be initiated at all) this belonged to.^26^ Moreover, linguistic aspects were assessed regarding recommendations to distinguish whether they were presented as a recommendation or as a decision. A recommendation was coded as a recommendation if word choice (e.g., explicit designation as a recommendation) or grammar (e.g., use of the subjunctive) indicated this. Conversely, it was coded as a decision if the corresponding word choice or grammar (utterance in the indicative) clarified that the decision had already been made. The next step was to examine the content of the healthcare professionals' expressions in terms of decision‐making stages. The categories were first created deductively based on the three‐talk model.^41^ In the application of SDM, Elwyn et al.^41^ distinguished the following phases in the three‐talk model: (i) team talk, in which the healthcare professional informs the patient that a choice is pending, support is offered and goals are queried; (ii) option talk, in which information about options is provided and risks and benefits are discussed and (iii) decision talk, in which a decision is made considering the preferences. The content of the utterances was examined to determine the extent to which the utterances could be assigned to one of the phases. Thus, we used the three‐talk model in the sense of a deductive ordering system and assigned the utterances with respect to SDM. In addition, it was analysed what exactly the utterance contained, for example, whether advantages and/or disadvantages of the option were discussed during the decision talk so that based on the available material, we adapted all categories inductively and expanded both based on the literature. The final codes concerning the three‐talk phases of decision‐making are presented in the results section with examples. For descriptive analysis, we counted how many codes could be assigned to each of the three phases of decision‐making.

## 3 RESULTS

### 3.1 Sample

Our study sample comprised *n* = 95 patients from four hospitals. Audio data from case discussions with patient participation were available for 82 patients.

#### 3.1.1 Sociodemographic and clinical characteristics of patients

Table 1 presents the sociodemographic and clinical characteristics of the study sample.

**Table 1:** Patients' characteristics (*n* = 82)

	*N*	%
**Age (years)**		
Mean (SD)	59 (11.2)	
**Marital status**		
Married	49	59.8
Widowed	9	11.0
Single	9	11.0
Divorced	8	9.8
Missing	7	8.5
**Children**		
Yes	61	74.4
No	15	18.3
Missing	6	7.3
**Living with partner**		
Yes	52	63.4
No	23	28.0
Missing	7	8.5
**Highest education level achieved**		
No lower secondary school education	1	1.2
Lower secondary school education	18	22.0
Intermediate secondary school education	23	28.0
University entrance certificate	32	39.0
Other	1	1.2
Missing	7	8.5
**Cancer entity**		
Breast cancer	77	93.9
Breast and gynaecologic cancer	5	6.1
**UICC stage**		
0	10	12.2
I	38	46.3
II	16	19.5
III	3	3.7
IV	10	12.2
Missing	5	6.1

#### 3.1.2 Case discussions in MTCs

The total length of audio data was 10:09:12 h, and the individual case discussions with attendance lasted between 01:25 and 20:54 min (median: 06:15 min). An average of eight healthcare professionals were present (range: 3–19) in MTCs. The seating arrangement of MTCs with participation was either theatre‐style (*n* = 5, 6%), U‐shaped (*n* = 17, 21%) or roundtable (*n* = 60, 73%). In five case discussions, patients were present during the entire case discussion. A total of 27 case discussions initially took place without patients, who were allowed to participate after the treatment recommendation was finalized. In addition, 50 case discussions initially took place without patients, and after the treatment recommendation was finalized, the discussion with the patients took place with fewer healthcare professionals in a separate room.

### 3.2 Decision‐making in MTCs with patient participation

#### 3.2.1 Addressing patients

During the analysis, it became apparent that there were parts of the conversation where the patients were present, but rather as observers. We observed two situations: (i) the patients were present from the beginning, but the healthcare professionals discussed the recommendations in the first part without addressing the patients, who were included in the conversation later; (ii) the healthcare professionals clarified points among themselves in the middle of the conversation with the patients but without addressing them.

#### 3.2.2 Recommendations

The presentation of the transcripts is explained in the following text to facilitate understanding. An accentuation is indicated by capital letters. Pauses within the utterance are shown in parentheses with dashes, depending on their length. Numbers in parentheses at the end of an utterance refer to the transcript number.

When recommendations were given, healthcare professionals referred to preliminary meetings where the likely recommendation had already been discussed.

And, um, it's been discussed in advance that if you preSERVE the breast, follow‐up radiotherapy in the region (‐) of the breast is necessary. (422)

Similarly, healthcare professionals referred to subsequent meetings, in which the general treatment course and the benefits and risks were to be described.

And that's why I would, um, recommend that you return to our office in the next few days so we can discuss with you the benefits and drawbacks and the BENEFIT you have from the late radiotherapy, so talk about everything, well, in peace and quiet. (645)

This was done by highlighting that the MTC is not the place for this detailed discussion.

Right now, we're just not in the right setting to talk about it in detail. (508)

Recommendations indicated that healthcare professionals' recommendations were based on the clinical practice guidelines.

Because (‐) there are guidelines. And if I depart from them and something happens to you and in three years, you come back to me and say: ‘Well, you said I don't have to’, then I made a mistake. So, I have to tell you, I have to recommend it to you. (402)

The recommendations presented in the case discussions could be categorized into the following types: the treatment, the time of starting the therapy, the next (treatment) steps or whether a treatment method should be initiated at all. In the cases analysed, it did not occur that no therapy was recommended, but individual therapy methods were excluded as options. While there were recommendations framed as a decision, others could not yet be provided, as they depended on the pending results or the further course of treatment. Moreover, we coded when healthcare professionals explicitly suggested that the decision about the recommendation depended on the patients' wishes. Table 2 presents the different types of recommendations with examples.

**Table 2:** Different types of recommendations with examples

Recommendations	
Recommendation (for treatment method)	HCP: We would recommend for you to have anti‐hormone therapy. (416)
Recommendation for (next) treatment steps	HCP: In terms of the sequence, it's always chemotherapy before radiotherapy. So, if chemotherapy is to be done, chemotherapy takes place before radiotherapy. (523)
Recommendation for treatment start/period	HCP: Radiotherapy would then take three weeks, 16 appointments. (510)
Recommendation/decision against treatment method	HCP: And um … Ultimately, chemotherapy wouldn't be recommended in this situation. (416)
Recommendation in the sense of decision	HCP: So the follow‐up treatment will then consist OF, um, taking an anti‐hormone tablet, (–) tamoxifen, for five years. (640)
Decision about the recommendation depending on further course/results	HCP: We'd have to initiate that, then. As soon as the results are in (‐) we'll know whether the next step is radiotherapy or anti‐hormone therapy or whether chemotherapy will be done too. (450)
Decision (about recommendation) depending on patients' wishes	HCP: Because if you say that I don't want it no matter what, it makes no sense to request such a test. Um, if you say, no, if it is recommended, I'd do it, then it makes sense to take the test, too. (523)

Abbreviation: HCP, healthcare professional.

#### 3.2.3 Three‐talk phases of decision‐making

##### Frequency of categories concerning the three‐talk phases

**Figure 1:**
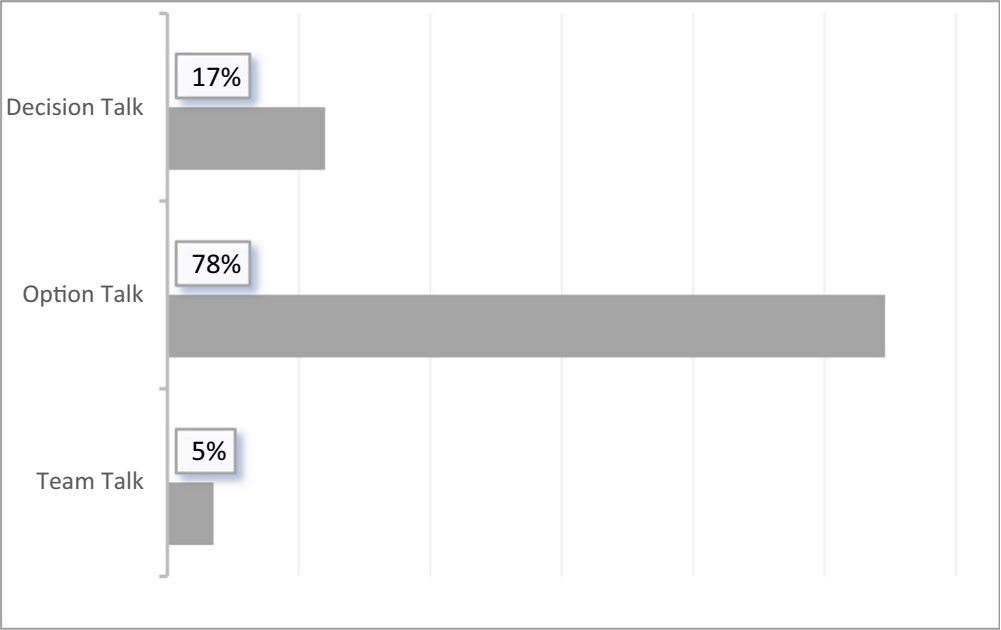
Percentage of utterances that could be assigned to the respective phases of the three‐talk phases

Figure 1 shows that the expressions in the case discussions were mainly attributed to option talk (78%), while decision talk (17%) and team talk (5%) accounted for a smaller share.

##### Team talk

In the case of discussions, we found individual elements of team talk. For example, it was highlighted that a decision must be made. However, healthcare professionals did not explicitly offer patients to participate in the conversation. Regarding the roles, the healthcare professionals emphasized that they (must) make a recommendation, that they can advise patients and that the decision ultimately remains with the patients. However, they also emphasized joint discussion and decision‐making as the process continued.

##### Option talk

A variety of categories are attributable to option talk. Healthcare professionals listed one or sometimes two possible options. However, patients were also told which (therapy) option the healthcare professionals did not recommend; that is, it was not an option for patients. Regarding different options, the option itself, the procedure, advantages and disadvantages were explained. In addition, disadvantages, such as side effects, were mentioned without further clarification. It occurred as well that patients were informed that they could decide against the recommendation, that is, not undergo the recommended treatment. For this purpose, disadvantages were listed that could occur if the patients refused the treatment; however, advantages were not listed in this regard. At this stage of the conversation, patients' possible psychosocial factors or comorbidities were also inquired. Furthermore, patients were asked whether they understood everything or whether they had any queries, although there was no comprehension check.

##### Decision talk

Preferences could be clarified and decisions could be made during case discussions. However, healthcare professionals could also highlight that the decision could be made at a later time. Moreover, the healthcare professionals explained the further procedure related to both the treatment and further organization and the upcoming process. Table 3 shows the extended category system with regard to the three‐talk model.

**Table 3:** The three‐talk phases of decision‐making

Phases of decision‐making	
**Team talk**	
Content of discussion	HCP: And now, for us, the question is what ELSE are we going to do for follow‐up treatment? (402)
Necessity of decision	HCP:/Basically, it's no use//discussing every detail, um, you have to make a decision. (402)
Role clarification	HCP: Well, I have to tell you, I have to (PA: Yes.) recommend it to you. But you can say: ‘I don't want that’. (402)
Equality	HCP: And then you will, I think, decide together whether you want to do that or not. (528)
Offer to participate in talk	
Offer of support/advice	HCP: Tomorrow, we'll take time to discuss everything. We'll have a lot of time. (419)
**Option talk**	
Naming of two (treatment) options	HCP: One can start right away now … Or (‐) wait for radiotherapy and start afterward. (640)
Naming of one (treatment) option	HCP: Now we also recommend that you have RADIOTHERAPY of the breast. (570)
Reason for (treatment) option	HCP: Then, as mentioned, radiotherapy of the breast on the left side and also the lymph drainage pathways because of these two lymph nodes that were involved, too. (506)
Explanation of treatment option	HCP: That basically catches the hormones from the body. (417)
Explanation of treatment procedure	HCP: Exactly, and then it'll be 28 therapies, right? So, almost six weeks, where you have to show up once every working day for radiotherapy. The procedure is a bit like taking an X‐ray, totally quick, really. You don't feel it at all. (530)
Benefits/advantages of option	HCP: It REDUCES the risk of recurrence. But, of course, never down to ZERO. Right? So, that's why, I'd say, um, you can't, um, fully answer that now. Right? Like I said, it's supposed to, um, (‐) help you to make sure that it doesn't recur. (509)
Risks/disadvantages of option	HCP: Unfortunately, it then goes to your bones, though. (618)
Mention of side effects	HCP: Of course, it's not a therapy that's free of side effects. (402)
Naming of nonoption	HCP: Antibody//therapy is not an option AT ALL. (443)
Reason for nonoption	HCP: … because the tumour was small, no lymph nodes involved, everything completely resected. And no gland tissue is really there any longer, either, right? (568)
Benefits/advantages of nonoption	HCP: Um, if you'd had no positive lymph nodes, we would have had the option of determining, through other tests, how well chemo can REALLY work; we CAN'T do that in your case. (530)
Risks/disadvantages of nonoption	HCP: But really, it's known not to help too much. Right? (PA: mhm) Traumatizes/injures the breast tissue even more, more than it actually helps. (638)
Naming of nonaction	HCP: Of course, things can work without this measure, too. (402)
Reason for nonaction	HCP: But actually, it's quite a favorable constellation of tumour tissue so that you might NOT need chemotherapy. (418)
Benefits/advantages of nonaction	
Risks/disadvantages of nonaction	HCP: And unfortunately, time and again, we see cases where patients decided against it and then come back in, right, because something recurred. (549)
Comprehension check	
Question for understanding	HCP: You know? (443)
Question about questions	HCP: (‐) Exactly. (‐) Any other questions? (805)
Psychosocial factors/comorbidities/pretreatment	HCP: Are you receiving psychiatric treatment? (546)
Ask about expectations, fears	HCP: While asking questions, I just said, when I put myself in your position now, after what, because we just … about the lungs … Then I would expect you to worry unnecessarily. (PA: I do. I did. I did.) That's what I thought. Yes. Do you still? (527)
**Decision talk**	
Clarification of preferences	HCP: I think I'm hearing that chemotherapy is NOT really an option (laughs) for you. (530)
Joint selection of treatment	PA: Yes. Yes. (‐) Yes, well, I mean, I have, I have decided for myself that whatever needs to be done (‐) I'll DO. HCP: You do want to do that, right? (742)
Postponement of decision	HCP: There's no need to make the decision here and now. You can think about it some more. (530)
Summary	HCP: So then we've integrated all elements for you. So that, let's say, we'd have you go through the trouble of taking a tablet initially, so to speak. (‐) Right? This aromatase inhibitor. It's the anti‐hormonal therapy. And then we wouldn't be able take the breast scan right away with the MRI but would try to, um, sit out this waiting period of six to eight weeks. (–) And once we have THAT, the MRI results, then, if there are no further findings, then Dr. (*name of HCP*) could start radiotherapy, too. (401)
Further procedure—Treatment	HCP: Promptly. So, we'll, as soon as possible, see to it that we do surgery on you either this week or next week at the latest. (418)
Further procedure—Organization	HCP: Exactly, then you're WELCOME to already schedule an appointment for a preliminary consultation. (561)

Abbreviations: HCP, healthcare professional; PA, patient.

## 4 DISCUSSION

This study gained insights into how recommendations are communicated to patients and the extent to which the interaction with patients in MTCs are in line with SDM. In the MTC, healthcare professionals make recommendations to patients regarding treatment options, treatment initiation, next (treatment) steps and whether a treatment method should be initiated at all. Recommendations were framed as both a recommendation and a final decision. During the decision‐making conversation, option talk predominantly occurred (78%), in which one (treatment) option was suggested to patients.

As the first step, healthcare professionals discuss the case and make a decision about their recommendations for patients without patient participation or involvement. Occasionally, healthcare professionals talk about patients in their presence without involving them at all. The presence of patients increased the participation, but they were not involved; this type of participation—being present during the discussion among healthcare professionals without being involved—could be helpful for understanding,^35^ or could lead to increased anxiety or stress.^8^, ^38^

While there are types of recommendations which reflect elements of SDM,^26^ other recommendations, framed as a decision, close the space for decision‐making and, thus, rather correspond to the paternalistic model. The survey data revealed that healthcare professionals perceive the opinions of MTCs as a decision in one‐quarter of cases,^23^ which is attributable to the clinical practice guidelines that limit the options in MTC,^39^ such as the recommendation for radiation after breast‐conserving surgery.^42^ In addition, our findings revealed that the recommendations were partly made with reference to the guideline based on the clinical findings, in line with a previous study^39^; this limits the choice of conceivable recommendations for healthcare professionals, but the decision to follow these recommendations lies with patients. As the majority of healthcare professionals also view the recommendation as such,^23^ MTCs could be used to draw attention to choice options. In addition, healthcare professionals emphasized during the team talk that they were providing recommendations, but patients had to finally make the decision, which reflected the informed model. We further specified the types of recommendation similar to the literature,^26^ when recommendations are promised, but the decision about the recommendation depends on patients' wishes, who are still given time to think about it, or also on pending results or the further course of treatment.

Elements of the three phases of the three‐talk model could be found in the case discussions, even if they did not always occur as defined by SDM as one of the three ideal types of models of decision‐making. For example, in team talk, roles are clarified, but often not in the sense of a conversation at eye level. The mere mention of an option is also not part of the option talk according to the definition of the SDM model. Most case discussions mainly included option talk, which seldom covered two equivalent options, rather the presentation of one option to patients, corroborating other studies demonstrating that patients are often presented with only one treatment option, which they can reject or accept.^43^ However, in professional practice, healthcare professionals use variations and hybrids when dealing with individual patients.^44^ Perhaps in MTCs, naming one option with pros and cons may represent the greatest possible patient participation in decision‐making in many cases. In an interview study, healthcare professionals emphasized that the alternative option was to opt out of the recommended treatment, that is, not undergo a recommended treatment.^39^ The option of doing nothing is also included in the International Standards for Patient Aid Standards.^45^ When patients understand the consequences of not receiving treatment, this understanding can assist in the decision to receive treatment^15^; however, this option is rarely explicitly stated in case discussions with patients nor is it presented with advantages and disadvantages. Moreover, in most cases, it is neglected to communicate that a decision is pending. During the option talk, healthcare professionals ask if patients have any queries or if they understand everything; at this point, however, no comprehension check is performed in the sense of teach‐back, which can be used as a method to further understanding.^46^

In the decision‐making process charted around MTCs without patient participation,^47^ the case discussion is only an intermediate step before the decision is subsequently made. Throughout the process, several discussions occur in different constellations. Even with patient participation, the case discussion in MTCs reflects only a small segment of the decision‐making process for different options; this is made clear as healthcare professionals refer to both prior and subsequent individual conversations with the patient. Furthermore, preliminary discussions might provide clues to the possible diagnosis^39^ or even already‐made treatment decisions that decrease the scope for decision‐making based on the clinical practice guidelines for further treatment.

In the subsequent discussions, for example, treatment methods are to be elucidated and the risks and side effects are to be explained. However, the reference to subsequent discussions is often accompanied by the comment that there is insufficient space for such information in the MTC owing to the lack of time, which, in turn, is also repeatedly highlighted as a disadvantage of patient participation in MTCs by healthcare professionals in other studies.^8^, ^38^ As recommendations are not implemented, in part, because of a lack of patient‐related factors,^14^, ^31^, ^32^, ^33^ the case discussion with participation could be used to obtain precisely this information. However, a preliminary study demonstrated that only about one‐third of the questions directed to patients in the MTC were used to obtain information.^48^

### 4.1 Strengths

To the best of our knowledge, the PINTU study is the first study to explore MTCs with patient participation. During the visit to the hospitals and MTCs before data collection, the research team explained the observation method^49^ to mitigate potential confounding factors related to the observer's presence and technical equipment for recording. Of note, observation and audio recordings are the major strengths of this study. We can now gain insights into the practice through observations^50^ and the recordings.

### 4.2 Limitations

A limitation of this study is that we did not collect data on the entire decision‐making process. Thus, we could not assess how much information patients received beforehand. The communication of choices could have occurred before the MTC, including outside of the face‐to‐face interview, via email or the provision of informational materials on the options.^51^ In addition, we did not observe the next steps of decision‐making. Hence, a study is warranted that follows patients and their decisions over the entire course of treatment to observe and assess the full range of decisions. Nevertheless, our study demonstrated what content regarding decision‐making can already be addressed in case discussions and what content does not currently seem to have space. In addition, there is a lack of evidence so far on how patients experience the decision‐making in the MTCs and how they evaluate the different implementation of participation in the centres: We have seen that patients sometimes participate but the conversation is not addressed to them. The question arises whether patients themselves evaluate this experience as participation or as observation.

## 5 CONCLUSIONS

The goal of MTCs involving patients does not seem to be making decisions but conveying recommendations that healthcare professionals have decided alone in advance. Patients can be prepared for this typical procedure in advance, for example, by breast care nurses. At the beginning of the discussion, healthcare professionals can highlight available choices in the decision‐making process to create an awareness of choices among patients.^43^ They often refer to debriefings where the pros and cons of the recommended treatments are explicitly discussed. At this point, it could be conveyed that a decision against treatment is also feasible. While the MTC is primarily about cancer and biomedical information, the discussion with patients following the MTC could focus on the patients similar to a patient board/patient conference.

## AUTHOR CONTRIBUTIONS

Barbara Schellenberger interpreted the results, drafted and revised all sections of the manuscript and is the guarantor. Christian Heuser, Annika Diekmann, Nicole Ernstmann and Lena Ansmann developed the study framework. Barbara Schellenberger, Christian Heuser and Annika Diekmann observed the MTCs. Barbara Schellenberger, Christian Heuser and Anna Schippers planned and conducted the data analysis. Christian Heuser, Nicole Ernstmann, Franziska Geiser, Ingo G. H. Schmidt‐Wolf, Isabelle Scholl and Lena Ansmann assisted in data interpretation and review and editing of the manuscript. All authors read and approved the final manuscript.

## CONFLICT OF INTEREST

Isabelle Scholl has been member of the executive board of the International Shared Decision Making Society, which has the mission to foster SDM implementation. The other authors declare no conflict of interest.

## ETHICS STATEMENT

This study was conducted per the Declaration of Helsinki and approved by the Ethics Committee of the Medical Faculty of the University of Cologne, Germany (Reference number for approval: 17‐405). Informed consent was obtained from all subjects involved in this study.

## Data Availability

The data that support the findings of this study are available on request from the corresponding author. The data are not publicly available due to privacy or ethical restrictions.
